# 3-(Adamantan-1-yl)-4-[(*E*)-(2,6-difluoro­benzyl­idene)amino]-1-[(4-phenyl­piperazin-1-yl)meth­yl]-1*H*-1,2,4-triazole-5(4*H*)-thione

**DOI:** 10.1107/S1600536812025135

**Published:** 2012-06-13

**Authors:** Ali A. El-Emam, Ebtehal S. Al-Abdullah, Nasser R. El-Brollosy, Seik Weng Ng, Edward R. T. Tiekink

**Affiliations:** a Department of Pharmaceutical Chemistry, College of Pharmacy, King Saud University, Riyadh 11451, Saudi Arabia; b Department of Chemistry, University of Malaya, 50603 Kuala Lumpur, Malaysia; c Chemistry Department, Faculty of Science, King Abdulaziz University, PO Box 80203 Jeddah, Saudi Arabia

## Abstract

The imine residue [C=N = 1.268 (3) Å; conformation = *E*] is twisted [N—N—C—N = 87.8 (2)°] out of the plane (r.m.s. deviation = 0.016 Å) of the central 1,2,4-triazole ring in the title compound, C_30_H_34_F_2_N_6_S. A small twist also occurs between the imine and terminal benzene rings [N—C—C—C = −169.8 (2)°]. The piperazine ring (chair conformation) occupies a position almost normal to the central plane [N—N—C—N = 87.8 (2)°]. In the crystal, the mol­ecules are consolidated into a three-dimensional architecture *via* C—H⋯S, C—H⋯π and π–π inter­actions, the latter between centrosymmetrically related difluoro­benzene rings [inter-centroid distance = 3.9389 (18) Å].

## Related literature

For a related structure and background to the biological activity of adamantane derivatives, see: El-Emam *et al.* (2012 ▶). For further synthetic details, see: Al-Omar *et al.* (2010 ▶).
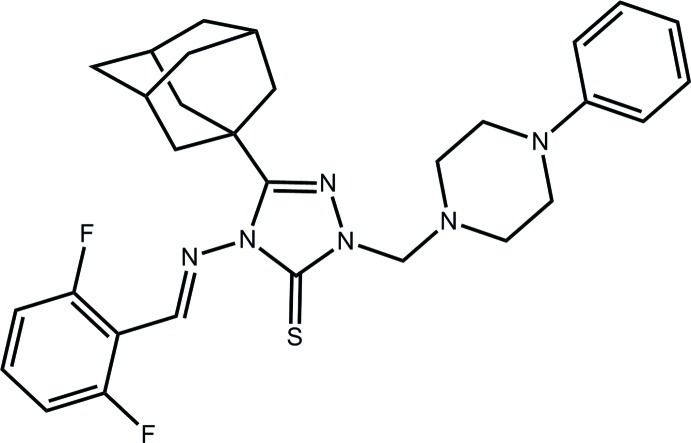


## Experimental

### 

#### Crystal data

C_30_H_34_F_2_N_6_S
*M* *_r_* = 548.69
Monoclinic, 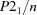

*a* = 17.2712 (3) Å
*b* = 7.7141 (1) Å
*c* = 21.3157 (4) Å
β = 95.245 (2)°
*V* = 2828.04 (8) Å^3^
*Z* = 4
Cu *K*α radiation
μ = 1.38 mm^−1^
*T* = 294 K
0.35 × 0.30 × 0.25 mm

#### Data collection

Agilent SuperNova Dual diffractometer with Atlas detector
Absorption correction: multi-scan (*CrysAlis PRO*; Agilent, 2012 ▶) *T*_min_ = 0.702, *T*_max_ = 1.000
20801 measured reflections
5884 independent reflections
4712 reflections with *I* > 2σ(*I*)
*R*_int_ = 0.027

#### Refinement

*R*[*F* ^2^ > 2σ(*F* ^2^)] = 0.053
*wR*(*F* ^2^) = 0.161
*S* = 1.04
5884 reflections
352 parameters
H-atom parameters constrained
Δρ_max_ = 0.70 e Å^−3^
Δρ_min_ = −0.42 e Å^−3^

### 

Data collection: *CrysAlis PRO* (Agilent, 2012 ▶); cell refinement: *CrysAlis PRO*; data reduction: *CrysAlis PRO*; program(s) used to solve structure: *SHELXS97* (Sheldrick, 2008 ▶); program(s) used to refine structure: *SHELXL97* (Sheldrick, 2008 ▶); molecular graphics: *ORTEP-3* (Farrugia, 1997 ▶) and *DIAMOND* (Brandenburg, 2006 ▶); software used to prepare material for publication: *publCIF* (Westrip, 2010 ▶).

## Supplementary Material

Crystal structure: contains datablock(s) global, I. DOI: 10.1107/S1600536812025135/hb6828sup1.cif

Structure factors: contains datablock(s) I. DOI: 10.1107/S1600536812025135/hb6828Isup2.hkl

Supplementary material file. DOI: 10.1107/S1600536812025135/hb6828Isup3.cml

Additional supplementary materials:  crystallographic information; 3D view; checkCIF report

## Figures and Tables

**Table 1:** Hydrogen-bond geometry (Å, °) *Cg*1 is the centroid of the C25–C30 ring.

*D*—H⋯*A*	*D*—H	H⋯*A*	*D*⋯*A*	*D*—H⋯*A*
C20—H20*B*⋯S1^i^	0.97	2.86	3.397 (2)	116
C28—H28⋯*Cg*1^ii^	0.93	2.99	3.832 (3)	151

Symmetry codes: (i) 
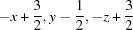
; (ii) 
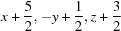
.

**Fig. 1.**
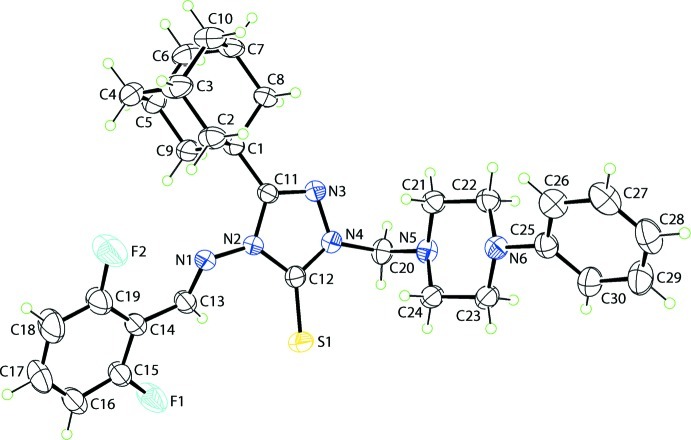
The molecular structure of (I) showing displacement ellipsoids at the 35% probability level.

**Fig. 2.**
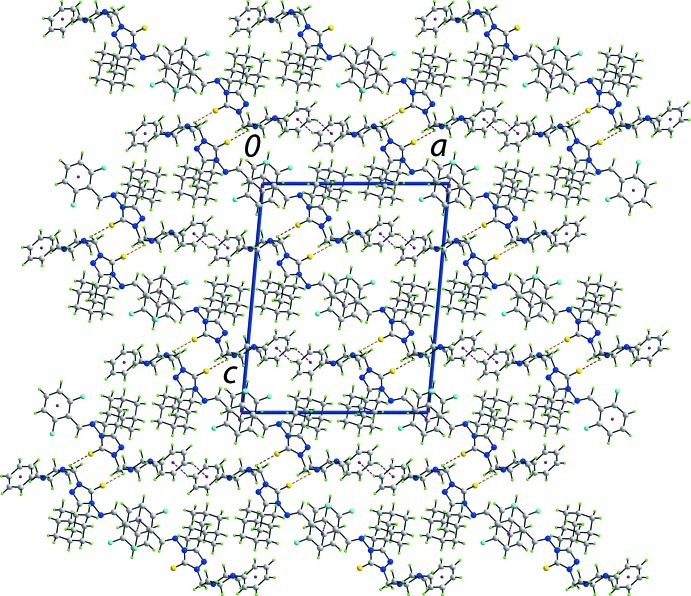
A view in projection down the b axis of the unit-cell contents for (I). The C—H···S, C—H···π and π—π interactions are shown as orange, purple and brown dashed lines, respectively.

## supplementary crystallographic information

### Comment

In continuation to our interest in the chemical and pharmacological properties of adamantane derivatives, and as part of on-going structural studies (El-Emam *et al.*, 2012), the title compound (I) was synthesized as potential chemotherapeutic agent (Al-Omar *et al.*, 2010).

In (I), the central 1,2,4-triazole ring (r.m.s. deviation = 0.016 Å) is twisted with respect to the adjacent imine bond (1.268 (3) Å; conformation = *E*) as seen in the value of the C13—N1—N2—C11 torsion angle of 148.49 (19)°. There is a small twist between the latter and the connected benzene ring with the N1—C13—C14—C15 torsion angle being -169.8 (2)°. The piperazine ring (chair conformation) projects nearly normal to the central plane [N3—N4—C20—N5 = 87.8 (2)°].

Molecules are consolidated in the crystal packing by a combination of C—H···S and C—H···π interactions, Table 1, as well as weak π—π interactions between centrosymmetrically related C(14—C19) benzene rings [inter-centroid distance = 3.9389 (18) Å for 1 - *x*, 1 - *y*, 1 - *z*], Fig. 2.

### Experimental

A mixture of the 5-(adamantan-1-yl)-4-(2,6-difluorobenzylideneamino)-4*H*-1,2,4-triazole-3-thiol (347 mg, 1 mmol), 1-phenylpiperazine (162 mg, 1 mmol) and 37% formaldehyde solution (0.5 ml), in ethanol (8 ml), was heated under reflux for 15 min. after which a clear solution was obtained. Stirring was continued for 12 h at room temperature and the mixture was allowed to stand overnight. Cold water (5 ml) was added and the mixture was stirred for a further 20 min. The precipitated crude product was filtered, washed with water, dried, and crystallized from ethanol to yield 434 mg (79%) of the title compound (I) as crystals. *M*.pt: 424–426 K. Light yellow prisms were obtained by slow evaporation of CHCl_3_:EtOH (1:1; 5 ml) solution at room temperature. ^1^H NMR (DMSO-d_6_, 500.13 MHz): δ 1.80 (s, 6H, adamantane-H), 2.10 (s, 3H, adamantane-H), 2.19 (s, 6H, adamantane-H), 3.04 (s, 4H, piperazine-H), 3.23 (s, 4H, piperazine-H), 5.24 (s, 2H, CH_2_), 6.89 (t, 1H, Ar—H, *J* = 7.0 Hz), 6.94 (d, 2H, Ar—H, *J* = 8.0 Hz), 7.03 (t. 2H, Ar—H, *J* = 8.5 Hz), 7.26–7.28 (m, 2H, Ar—H), 7.47–7.50 (m. 1H, Ar—H), 10.67 (s, 1H, CH=N). ^13^C NMR (DMSO-d_6_, 125.76 MHz): δ 28.0, 35.56, 36.46, 38.37 (adamantane-C), 49.41, 50.55 (piperazine-C), 68.82 (CH_2_), 110.76, 112.19, 116.31, 119.88, 129.10, 133.27, 151.38, 152.24 (Ar—C), 155.64, 161.06 (triazole C-5 & CH=N), 163.22 (C=S).

### Refinement

Carbon-bound H-atoms were placed in calculated positions [C—H = 0.93 to 0.98 Å, *U*_iso_(H) = 1.2*U*_eq_(C)] and were included in the refinement in the riding model approximation.

### Crystal data

**Table:**

C_30_H_34_F_2_N_6_S	*F*(000) = 1160
*M**_r_* = 548.69	*D*_x_ = 1.289 Mg m^−^^3^
Monoclinic, *P*2_1_/*n*	Cu *K*α radiation, λ = 1.54184 Å
Hall symbol: -P 2yn	Cell parameters from 6884 reflections
*a* = 17.2712 (3) Å	θ = 3.2–76.4°
*b* = 7.7141 (1) Å	µ = 1.38 mm^−^^1^
*c* = 21.3157 (4) Å	*T* = 294 K
β = 95.245 (2)°	Prism, light-yellow
*V* = 2828.04 (8) Å^3^	0.35 × 0.30 × 0.25 mm
*Z* = 4	

### Data collection

**Table:**

Agilent SuperNova Dual diffractometer with Atlas detector	5884 independent reflections
Radiation source: SuperNova (Cu) X-ray Source	4712 reflections with *I* > 2σ(*I*)
Mirror monochromator	*R*_int_ = 0.027
Detector resolution: 10.4041 pixels mm^-1^	θ_max_ = 76.6°, θ_min_ = 3.2°
ω scan	*h* = −21→11
Absorption correction: multi-scan (*CrysAlis PRO*; Agilent, 2012)	*k* = −9→9
*T*_min_ = 0.702, *T*_max_ = 1.000	*l* = −24→26
20801 measured reflections	

### Refinement

**Table:**

Refinement on *F*^2^	Primary atom site location: structure-invariant direct methods
Least-squares matrix: full	Secondary atom site location: difference Fourier map
*R*[*F*^2^ > 2σ(*F*^2^)] = 0.053	Hydrogen site location: inferred from neighbouring sites
*wR*(*F*^2^) = 0.161	H-atom parameters constrained
*S* = 1.04	*w* = 1/[σ^2^(*F*_o_^2^) + (0.0841*P*)^2^ + 0.8642*P*] where *P* = (*F*_o_^2^ + 2*F*_c_^2^)/3
5884 reflections	(Δ/σ)_max_ < 0.001
352 parameters	Δρ_max_ = 0.70 e Å^−^^3^
0 restraints	Δρ_min_ = −0.42 e Å^−^^3^

### Special details

**Table:**

Geometry. All e.s.d.'s (except the e.s.d. in the dihedral angle between two l.s. planes) are estimated using the full covariance matrix. The cell e.s.d.'s are taken into account individually in the estimation of e.s.d.'s in distances, angles and torsion angles; correlations between e.s.d.'s in cell parameters are only used when they are defined by crystal symmetry. An approximate (isotropic) treatment of cell e.s.d.'s is used for estimating e.s.d.'s involving l.s. planes.
Refinement. Refinement of *F*^2^ against ALL reflections. The weighted *R*-factor *wR* and goodness of fit *S* are based on *F*^2^, conventional *R*-factors *R* are based on *F*, with *F* set to zero for negative *F*^2^. The threshold expression of *F*^2^ > σ(*F*^2^) is used only for calculating *R*-factors(gt) *etc*. and is not relevant to the choice of reflections for refinement. *R*-factors based on *F*^2^ are statistically about twice as large as those based on *F*, and *R*- factors based on ALL data will be even larger.

### Fractional atomic coordinates and isotropic or equivalent isotropic displacement parameters (Å^2^)

**Table:**

	*x*	*y*	*z*	*U*_iso_*/*U*_eq_	
S1	0.71076 (3)	0.89349 (8)	0.68065 (3)	0.06427 (19)	
N1	0.70514 (9)	0.6526 (2)	0.55097 (8)	0.0518 (4)	
N2	0.76034 (9)	0.6366 (2)	0.60276 (8)	0.0454 (4)	
N3	0.87099 (9)	0.5541 (2)	0.65262 (8)	0.0471 (4)	
N4	0.83637 (9)	0.6826 (2)	0.68561 (8)	0.0479 (4)	
N5	0.93244 (10)	0.8605 (2)	0.75009 (8)	0.0501 (4)	
N6	1.03325 (10)	1.1263 (2)	0.71401 (8)	0.0502 (4)	
F1	0.49418 (9)	0.7917 (3)	0.59319 (7)	0.1000 (6)	
F2	0.64051 (11)	0.6213 (4)	0.43092 (8)	0.1399 (11)	
C1	0.83845 (10)	0.3955 (2)	0.55331 (9)	0.0443 (4)	
C2	0.84385 (14)	0.4736 (3)	0.48742 (10)	0.0599 (5)	
H2A	0.7959	0.5337	0.4738	0.072*	
H2B	0.8862	0.5566	0.4888	0.072*	
C3	0.85804 (18)	0.3275 (4)	0.44079 (11)	0.0759 (8)	
H3	0.8606	0.3768	0.3987	0.091*	
C4	0.79106 (18)	0.1981 (4)	0.43920 (15)	0.0944 (11)	
H4A	0.7985	0.1080	0.4086	0.113*	
H4B	0.7424	0.2567	0.4266	0.113*	
C5	0.78757 (15)	0.1172 (3)	0.50410 (15)	0.0760 (8)	
H5	0.7452	0.0325	0.5025	0.091*	
C6	0.86387 (16)	0.0277 (3)	0.52427 (14)	0.0707 (7)	
H6A	0.8729	−0.0644	0.4948	0.085*	
H6B	0.8617	−0.0234	0.5656	0.085*	
C7	0.92952 (13)	0.1579 (3)	0.52613 (11)	0.0577 (5)	
H7	0.9786	0.0994	0.5395	0.069*	
C8	0.91554 (11)	0.3024 (3)	0.57298 (10)	0.0511 (5)	
H8A	0.9580	0.3850	0.5744	0.061*	
H8B	0.9139	0.2536	0.6148	0.061*	
C9	0.77257 (13)	0.2608 (3)	0.55135 (13)	0.0616 (6)	
H9A	0.7700	0.2110	0.5929	0.074*	
H9B	0.7232	0.3165	0.5389	0.074*	
C10	0.93432 (16)	0.2354 (3)	0.46118 (12)	0.0699 (7)	
H10A	0.9436	0.1446	0.4313	0.084*	
H10B	0.9771	0.3172	0.4622	0.084*	
C11	0.82487 (10)	0.5307 (2)	0.60164 (9)	0.0437 (4)	
C12	0.76867 (11)	0.7375 (2)	0.65675 (9)	0.0467 (4)	
C13	0.63618 (11)	0.6808 (3)	0.56417 (10)	0.0531 (5)	
H13	0.6258	0.6842	0.6062	0.064*	
C14	0.57280 (11)	0.7082 (3)	0.51524 (10)	0.0519 (5)	
C15	0.50136 (12)	0.7655 (3)	0.53143 (11)	0.0598 (5)	
C16	0.43828 (14)	0.8010 (4)	0.48995 (13)	0.0734 (7)	
H16	0.3922	0.8421	0.5039	0.088*	
C17	0.44493 (16)	0.7743 (4)	0.42783 (13)	0.0836 (8)	
H17	0.4028	0.7972	0.3986	0.100*	
C18	0.51275 (19)	0.7141 (5)	0.40770 (13)	0.0967 (11)	
H18	0.5169	0.6949	0.3651	0.116*	
C19	0.57489 (15)	0.6823 (5)	0.45122 (12)	0.0794 (8)	
C20	0.87344 (13)	0.7298 (3)	0.74837 (9)	0.0529 (5)	
H20A	0.8332	0.7690	0.7739	0.064*	
H20B	0.8962	0.6260	0.7680	0.064*	
C21	0.99593 (13)	0.8207 (3)	0.71174 (11)	0.0560 (5)	
H21A	0.9773	0.8259	0.6675	0.067*	
H21B	1.0148	0.7042	0.7210	0.067*	
C22	1.06122 (12)	0.9487 (3)	0.72533 (11)	0.0566 (5)	
H22A	1.0825	0.9371	0.7688	0.068*	
H22B	1.1024	0.9241	0.6986	0.068*	
C23	0.96833 (13)	1.1645 (3)	0.75066 (11)	0.0582 (5)	
H23A	0.9493	1.2806	0.7409	0.070*	
H23B	0.9857	1.1601	0.7952	0.070*	
C24	0.90366 (12)	1.0356 (3)	0.73619 (11)	0.0546 (5)	
H24A	0.8611	1.0613	0.7614	0.066*	
H24B	0.8844	1.0439	0.6921	0.066*	
C25	1.09395 (12)	1.2508 (3)	0.71727 (10)	0.0525 (5)	
C26	1.14778 (15)	1.2456 (3)	0.67281 (12)	0.0653 (6)	
H26	1.1430	1.1623	0.6412	0.078*	
C27	1.20870 (15)	1.3638 (4)	0.67517 (14)	0.0754 (7)	
H27	1.2447	1.3573	0.6454	0.090*	
C28	1.21662 (16)	1.4893 (4)	0.72056 (15)	0.0796 (8)	
H28	1.2574	1.5683	0.7217	0.096*	
C29	1.16349 (17)	1.4969 (4)	0.76432 (16)	0.0864 (8)	
H29	1.1682	1.5821	0.7953	0.104*	
C30	1.10264 (15)	1.3788 (3)	0.76298 (13)	0.0705 (6)	
H30	1.0672	1.3857	0.7932	0.085*	

### Atomic displacement parameters (Å^2^)

**Table:**

	*U*^11^	*U*^22^	*U*^33^	*U*^12^	*U*^13^	*U*^23^
S1	0.0526 (3)	0.0593 (3)	0.0821 (4)	0.0057 (2)	0.0122 (3)	−0.0215 (3)
N1	0.0425 (8)	0.0561 (9)	0.0551 (9)	0.0104 (7)	−0.0041 (7)	−0.0122 (8)
N2	0.0376 (7)	0.0450 (8)	0.0528 (9)	0.0048 (6)	0.0006 (6)	−0.0087 (7)
N3	0.0449 (8)	0.0433 (8)	0.0526 (9)	0.0020 (6)	0.0018 (7)	−0.0036 (7)
N4	0.0469 (8)	0.0469 (8)	0.0496 (9)	−0.0021 (7)	0.0022 (7)	−0.0059 (7)
N5	0.0538 (9)	0.0519 (9)	0.0444 (8)	−0.0070 (7)	0.0029 (7)	−0.0015 (7)
N6	0.0504 (9)	0.0468 (9)	0.0533 (9)	−0.0008 (7)	0.0035 (7)	−0.0014 (7)
F1	0.0608 (9)	0.179 (2)	0.0605 (9)	0.0320 (10)	0.0098 (7)	−0.0038 (10)
F2	0.0842 (12)	0.269 (3)	0.0661 (10)	0.0617 (16)	0.0053 (8)	−0.0370 (14)
C1	0.0386 (9)	0.0411 (9)	0.0530 (10)	0.0057 (7)	0.0039 (7)	−0.0042 (8)
C2	0.0686 (13)	0.0560 (12)	0.0551 (12)	0.0226 (10)	0.0052 (10)	0.0066 (9)
C3	0.0965 (19)	0.0818 (17)	0.0489 (12)	0.0411 (16)	0.0042 (12)	−0.0001 (11)
C4	0.0862 (19)	0.103 (2)	0.088 (2)	0.0409 (18)	−0.0264 (15)	−0.0497 (18)
C5	0.0591 (13)	0.0621 (14)	0.106 (2)	−0.0035 (11)	0.0020 (13)	−0.0342 (14)
C6	0.0834 (17)	0.0453 (11)	0.0852 (17)	0.0111 (11)	0.0181 (13)	−0.0101 (11)
C7	0.0553 (12)	0.0524 (11)	0.0657 (13)	0.0215 (9)	0.0073 (10)	−0.0006 (9)
C8	0.0457 (10)	0.0487 (10)	0.0583 (11)	0.0123 (8)	0.0016 (8)	0.0008 (9)
C9	0.0464 (11)	0.0537 (11)	0.0856 (16)	−0.0028 (9)	0.0116 (10)	−0.0178 (11)
C10	0.0753 (15)	0.0689 (14)	0.0686 (14)	0.0246 (12)	0.0234 (12)	0.0017 (11)
C11	0.0358 (8)	0.0400 (9)	0.0552 (10)	0.0021 (7)	0.0037 (7)	−0.0018 (8)
C12	0.0416 (9)	0.0453 (9)	0.0536 (10)	−0.0048 (7)	0.0060 (8)	−0.0056 (8)
C13	0.0437 (10)	0.0663 (12)	0.0486 (10)	0.0059 (9)	0.0002 (8)	−0.0017 (9)
C14	0.0420 (10)	0.0614 (12)	0.0512 (11)	0.0073 (9)	−0.0018 (8)	−0.0035 (9)
C15	0.0465 (11)	0.0773 (15)	0.0548 (12)	0.0054 (10)	0.0005 (9)	−0.0012 (10)
C16	0.0440 (11)	0.0945 (19)	0.0794 (16)	0.0116 (12)	−0.0074 (11)	−0.0006 (14)
C17	0.0622 (15)	0.112 (2)	0.0715 (17)	0.0029 (15)	−0.0201 (12)	0.0101 (15)
C18	0.0803 (19)	0.155 (3)	0.0513 (14)	0.012 (2)	−0.0112 (12)	−0.0078 (17)
C19	0.0598 (14)	0.122 (2)	0.0553 (13)	0.0187 (15)	0.0008 (11)	−0.0115 (14)
C20	0.0589 (12)	0.0555 (11)	0.0440 (10)	−0.0103 (9)	0.0031 (8)	0.0012 (8)
C21	0.0549 (11)	0.0461 (10)	0.0672 (13)	0.0014 (9)	0.0068 (10)	−0.0027 (9)
C22	0.0515 (11)	0.0505 (11)	0.0674 (13)	0.0014 (9)	0.0035 (9)	0.0008 (9)
C23	0.0590 (12)	0.0504 (11)	0.0662 (13)	−0.0052 (9)	0.0113 (10)	−0.0125 (10)
C24	0.0531 (11)	0.0497 (11)	0.0622 (12)	−0.0033 (9)	0.0113 (9)	−0.0086 (9)
C25	0.0506 (11)	0.0503 (10)	0.0551 (11)	−0.0004 (8)	−0.0028 (9)	0.0073 (9)
C26	0.0664 (14)	0.0652 (14)	0.0647 (13)	−0.0024 (11)	0.0084 (11)	0.0098 (11)
C27	0.0595 (14)	0.0806 (17)	0.0866 (18)	−0.0024 (12)	0.0090 (13)	0.0292 (15)
C28	0.0610 (15)	0.0745 (17)	0.100 (2)	−0.0154 (13)	−0.0108 (14)	0.0199 (15)
C29	0.0782 (18)	0.0761 (17)	0.103 (2)	−0.0223 (15)	−0.0043 (16)	−0.0118 (16)
C30	0.0657 (14)	0.0648 (14)	0.0804 (16)	−0.0138 (11)	0.0038 (12)	−0.0098 (12)

### Geometric parameters (Å, º)

**Table:**

S1—C12	1.6735 (19)	C8—H8B	0.9700
N1—C13	1.268 (3)	C9—H9A	0.9700
N1—N2	1.397 (2)	C9—H9B	0.9700
N2—C11	1.384 (2)	C10—H10A	0.9700
N2—C12	1.386 (2)	C10—H10B	0.9700
N3—C11	1.300 (2)	C13—C14	1.457 (3)
N3—N4	1.382 (2)	C13—H13	0.9300
N4—C12	1.339 (3)	C14—C19	1.383 (3)
N4—C20	1.475 (2)	C14—C15	1.384 (3)
N5—C20	1.431 (3)	C15—C16	1.366 (3)
N5—C21	1.459 (3)	C16—C17	1.355 (4)
N5—C24	1.461 (3)	C16—H16	0.9300
N6—C25	1.419 (3)	C17—C18	1.365 (4)
N6—C23	1.454 (3)	C17—H17	0.9300
N6—C22	1.465 (3)	C18—C19	1.375 (4)
F1—C15	1.349 (3)	C18—H18	0.9300
F2—C19	1.335 (3)	C20—H20A	0.9700
C1—C11	1.500 (3)	C20—H20B	0.9700
C1—C8	1.537 (2)	C21—C22	1.507 (3)
C1—C2	1.539 (3)	C21—H21A	0.9700
C1—C9	1.539 (3)	C21—H21B	0.9700
C2—C3	1.537 (3)	C22—H22A	0.9700
C2—H2A	0.9700	C22—H22B	0.9700
C2—H2B	0.9700	C23—C24	1.506 (3)
C3—C4	1.526 (5)	C23—H23A	0.9700
C3—C10	1.525 (3)	C23—H23B	0.9700
C3—H3	0.9800	C24—H24A	0.9700
C4—C5	1.524 (5)	C24—H24B	0.9700
C4—H4A	0.9700	C25—C26	1.387 (3)
C4—H4B	0.9700	C25—C30	1.386 (3)
C5—C6	1.515 (4)	C26—C27	1.390 (4)
C5—C9	1.535 (3)	C26—H26	0.9300
C5—H5	0.9800	C27—C28	1.367 (4)
C6—C7	1.512 (4)	C27—H27	0.9300
C6—H6A	0.9700	C28—C29	1.368 (4)
C6—H6B	0.9700	C28—H28	0.9300
C7—C10	1.517 (3)	C29—C30	1.389 (4)
C7—C8	1.531 (3)	C29—H29	0.9300
C7—H7	0.9800	C30—H30	0.9300
C8—H8A	0.9700		
			
C13—N1—N2	115.28 (17)	N3—C11—C1	123.27 (16)
C11—N2—C12	108.85 (15)	N2—C11—C1	126.81 (16)
C11—N2—N1	122.08 (15)	N4—C12—N2	102.93 (16)
C12—N2—N1	128.21 (15)	N4—C12—S1	127.49 (15)
C11—N3—N4	105.32 (15)	N2—C12—S1	129.57 (15)
C12—N4—N3	113.15 (16)	N1—C13—C14	121.72 (19)
C12—N4—C20	128.81 (17)	N1—C13—H13	119.1
N3—N4—C20	117.80 (16)	C14—C13—H13	119.1
C20—N5—C21	114.03 (17)	C19—C14—C15	113.36 (19)
C20—N5—C24	114.70 (17)	C19—C14—C13	126.9 (2)
C21—N5—C24	109.89 (16)	C15—C14—C13	119.77 (19)
C25—N6—C23	116.06 (16)	F1—C15—C16	117.5 (2)
C25—N6—C22	113.18 (16)	F1—C15—C14	117.06 (19)
C23—N6—C22	111.08 (17)	C16—C15—C14	125.4 (2)
C11—C1—C8	108.87 (16)	C17—C16—C15	117.9 (2)
C11—C1—C2	112.39 (16)	C17—C16—H16	121.1
C8—C1—C2	107.97 (16)	C15—C16—H16	121.1
C11—C1—C9	109.08 (15)	C16—C17—C18	120.7 (2)
C8—C1—C9	108.18 (16)	C16—C17—H17	119.7
C2—C1—C9	110.24 (18)	C18—C17—H17	119.7
C3—C2—C1	109.26 (18)	C17—C18—C19	119.2 (3)
C3—C2—H2A	109.8	C17—C18—H18	120.4
C1—C2—H2A	109.8	C19—C18—H18	120.4
C3—C2—H2B	109.8	F2—C19—C18	118.5 (2)
C1—C2—H2B	109.8	F2—C19—C14	118.0 (2)
H2A—C2—H2B	108.3	C18—C19—C14	123.4 (2)
C4—C3—C10	109.6 (2)	N5—C20—N4	116.50 (16)
C4—C3—C2	109.2 (2)	N5—C20—H20A	108.2
C10—C3—C2	110.0 (2)	N4—C20—H20A	108.2
C4—C3—H3	109.4	N5—C20—H20B	108.2
C10—C3—H3	109.4	N4—C20—H20B	108.2
C2—C3—H3	109.4	H20A—C20—H20B	107.3
C3—C4—C5	109.9 (2)	N5—C21—C22	109.98 (18)
C3—C4—H4A	109.7	N5—C21—H21A	109.7
C5—C4—H4A	109.7	C22—C21—H21A	109.7
C3—C4—H4B	109.7	N5—C21—H21B	109.7
C5—C4—H4B	109.7	C22—C21—H21B	109.7
H4A—C4—H4B	108.2	H21A—C21—H21B	108.2
C6—C5—C4	109.8 (2)	N6—C22—C21	110.58 (17)
C6—C5—C9	109.7 (2)	N6—C22—H22A	109.5
C4—C5—C9	108.9 (2)	C21—C22—H22A	109.5
C6—C5—H5	109.5	N6—C22—H22B	109.5
C4—C5—H5	109.5	C21—C22—H22B	109.5
C9—C5—H5	109.5	H22A—C22—H22B	108.1
C5—C6—C7	109.6 (2)	N6—C23—C24	110.50 (17)
C5—C6—H6A	109.8	N6—C23—H23A	109.6
C7—C6—H6A	109.8	C24—C23—H23A	109.6
C5—C6—H6B	109.8	N6—C23—H23B	109.6
C7—C6—H6B	109.8	C24—C23—H23B	109.6
H6A—C6—H6B	108.2	H23A—C23—H23B	108.1
C6—C7—C10	110.0 (2)	N5—C24—C23	109.68 (18)
C6—C7—C8	109.76 (18)	N5—C24—H24A	109.7
C10—C7—C8	109.45 (19)	C23—C24—H24A	109.7
C6—C7—H7	109.2	N5—C24—H24B	109.7
C10—C7—H7	109.2	C23—C24—H24B	109.7
C8—C7—H7	109.2	H24A—C24—H24B	108.2
C7—C8—C1	110.28 (17)	C26—C25—C30	117.7 (2)
C7—C8—H8A	109.6	C26—C25—N6	119.0 (2)
C1—C8—H8A	109.6	C30—C25—N6	123.3 (2)
C7—C8—H8B	109.6	C25—C26—C27	120.6 (3)
C1—C8—H8B	109.6	C25—C26—H26	119.7
H8A—C8—H8B	108.1	C27—C26—H26	119.7
C5—C9—C1	109.66 (18)	C28—C27—C26	121.1 (3)
C5—C9—H9A	109.7	C28—C27—H27	119.4
C1—C9—H9A	109.7	C26—C27—H27	119.4
C5—C9—H9B	109.7	C27—C28—C29	118.9 (3)
C1—C9—H9B	109.7	C27—C28—H28	120.6
H9A—C9—H9B	108.2	C29—C28—H28	120.6
C7—C10—C3	109.02 (19)	C28—C29—C30	120.7 (3)
C7—C10—H10A	109.9	C28—C29—H29	119.6
C3—C10—H10A	109.9	C30—C29—H29	119.6
C7—C10—H10B	109.9	C29—C30—C25	121.0 (3)
C3—C10—H10B	109.9	C29—C30—H30	119.5
H10A—C10—H10B	108.3	C25—C30—H30	119.5
N3—C11—N2	109.67 (16)		
			
C13—N1—N2—C11	148.49 (19)	C11—N2—C12—N4	−1.8 (2)
C13—N1—N2—C12	−43.3 (3)	N1—N2—C12—N4	−171.19 (18)
C11—N3—N4—C12	1.3 (2)	C11—N2—C12—S1	176.97 (15)
C11—N3—N4—C20	176.19 (16)	N1—N2—C12—S1	7.5 (3)
C11—C1—C2—C3	179.79 (19)	N2—N1—C13—C14	177.22 (19)
C8—C1—C2—C3	59.7 (2)	N1—C13—C14—C19	10.4 (4)
C9—C1—C2—C3	−58.3 (2)	N1—C13—C14—C15	−169.8 (2)
C1—C2—C3—C4	59.4 (3)	C19—C14—C15—F1	179.2 (3)
C1—C2—C3—C10	−60.8 (3)	C13—C14—C15—F1	−0.7 (4)
C10—C3—C4—C5	58.8 (3)	C19—C14—C15—C16	−2.3 (4)
C2—C3—C4—C5	−61.7 (3)	C13—C14—C15—C16	177.9 (3)
C3—C4—C5—C6	−58.6 (3)	F1—C15—C16—C17	−179.7 (3)
C3—C4—C5—C9	61.6 (3)	C14—C15—C16—C17	1.7 (5)
C4—C5—C6—C7	59.2 (3)	C15—C16—C17—C18	−0.1 (5)
C9—C5—C6—C7	−60.4 (3)	C16—C17—C18—C19	−0.7 (6)
C5—C6—C7—C10	−60.6 (2)	C17—C18—C19—F2	179.1 (4)
C5—C6—C7—C8	59.9 (3)	C17—C18—C19—C14	0.0 (6)
C6—C7—C8—C1	−59.9 (2)	C15—C14—C19—F2	−177.8 (3)
C10—C7—C8—C1	60.9 (2)	C13—C14—C19—F2	2.0 (5)
C11—C1—C8—C7	177.53 (17)	C15—C14—C19—C18	1.4 (5)
C2—C1—C8—C7	−60.2 (2)	C13—C14—C19—C18	−178.8 (3)
C9—C1—C8—C7	59.1 (2)	C21—N5—C20—N4	−56.1 (2)
C6—C5—C9—C1	60.6 (3)	C24—N5—C20—N4	71.8 (2)
C4—C5—C9—C1	−59.6 (3)	C12—N4—C20—N5	−98.2 (2)
C11—C1—C9—C5	−177.6 (2)	N3—N4—C20—N5	87.8 (2)
C8—C1—C9—C5	−59.3 (3)	C20—N5—C21—C22	−170.14 (18)
C2—C1—C9—C5	58.6 (2)	C24—N5—C21—C22	59.5 (2)
C6—C7—C10—C3	60.6 (3)	C25—N6—C22—C21	−171.53 (18)
C8—C7—C10—C3	−60.0 (3)	C23—N6—C22—C21	55.8 (2)
C4—C3—C10—C7	−59.5 (3)	N5—C21—C22—N6	−57.1 (2)
C2—C3—C10—C7	60.5 (3)	C25—N6—C23—C24	172.32 (18)
N4—N3—C11—N2	−2.3 (2)	C22—N6—C23—C24	−56.5 (2)
N4—N3—C11—C1	−176.97 (16)	C20—N5—C24—C23	169.94 (17)
C12—N2—C11—N3	2.7 (2)	C21—N5—C24—C23	−60.0 (2)
N1—N2—C11—N3	172.89 (17)	N6—C23—C24—N5	58.5 (2)
C12—N2—C11—C1	177.08 (18)	C23—N6—C25—C26	−164.6 (2)
N1—N2—C11—C1	−12.7 (3)	C22—N6—C25—C26	65.2 (3)
C8—C1—C11—N3	−3.0 (3)	C23—N6—C25—C30	15.4 (3)
C2—C1—C11—N3	−122.6 (2)	C22—N6—C25—C30	−114.7 (2)
C9—C1—C11—N3	114.9 (2)	C30—C25—C26—C27	1.0 (3)
C8—C1—C11—N2	−176.68 (18)	N6—C25—C26—C27	−179.0 (2)
C2—C1—C11—N2	63.7 (2)	C25—C26—C27—C28	−1.0 (4)
C9—C1—C11—N2	−58.8 (3)	C26—C27—C28—C29	0.3 (4)
N3—N4—C12—N2	0.4 (2)	C27—C28—C29—C30	0.3 (5)
C20—N4—C12—N2	−173.89 (18)	C28—C29—C30—C25	−0.2 (5)
N3—N4—C12—S1	−178.41 (14)	C26—C25—C30—C29	−0.4 (4)
C20—N4—C12—S1	7.3 (3)	N6—C25—C30—C29	179.6 (2)

### Hydrogen-bond geometry (Å, º)

Cg1 is the centroid of the C25–C30 ring.

**Table:**

*D*—H···*A*	*D*—H	H···*A*	*D*···*A*	*D*—H···*A*
C20—H20*B*···S1^i^	0.97	2.86	3.397 (2)	116
C28—H28···*Cg*1^ii^	0.93	2.99	3.832 (3)	151

Symmetry codes: (i) −*x*+3/2, *y*−1/2, −*z*+3/2; (ii) *x*+5/2, −*y*+1/2, *z*+3/2.
